# 2-{1-[2-((2-Ammonio­ethyl){2-[1-(5-chloro-2-hydroxy­phen­yl)ethyl­idene­amino]eth­yl}amino)ethyl­iminio]eth­yl}-4-chloro­phenolate trifluoro­acetate

**DOI:** 10.1107/S1600536809002943

**Published:** 2009-01-28

**Authors:** See Mun Lee, Hapipah Mohd. Ali, Kong Mun Lo, Seik Weng Ng

**Affiliations:** a Department of Chemistry, University of Malaya, 50603 Kuala Lumpur, Malaysia

## Abstract

In the title ion-pair, C_22_H_29_Cl_2_N_4_O_2_^+^·C_2_F_3_O_2_^−^, ammonium–carboxyl­ate N—H⋯O hydrogen bonds link two cations and two anions about a centre of inversion to generate a hydrogen-bonded tetramer. In the cation, one of the imino N atoms is protonated and donates a hydrogen bond to the O atom of the adjacent chloro­phenyl ring. The other imino N atom acts as a hydrogen-bond acceptor from a phenolate O atom.

## Related literature

The precursor Schiff base, bis­{2-[1-(5-chloro-2-hydroxyphenyl)ethyl­eneamino]eth­yl}{2-[1-(5-chloro-2-phenolate)ethyl­ene­aminio]eth­yl}amine, has one of the three C=N double bonds protonated on the N atom (Lee *et al.*, 2009 ▶).
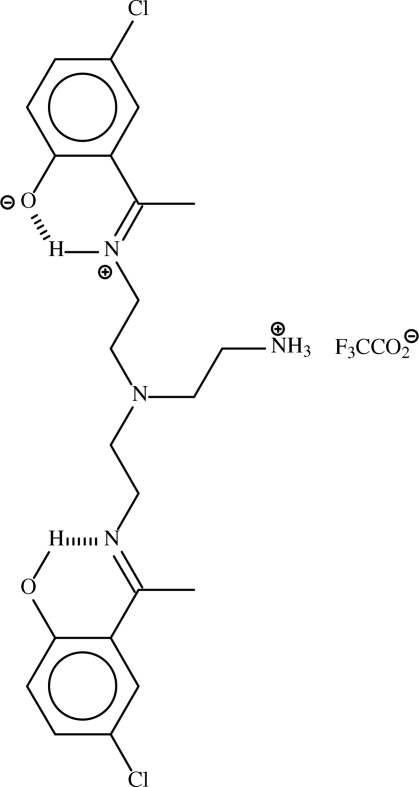


## Experimental

### 

#### Crystal data

C_22_H_29_Cl_2_N_4_O_2_^+^·C_2_F_3_O_2_^−^
*M* *_r_* = 565.41
Triclinic, 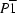

*a* = 10.1041 (2) Å
*b* = 10.6788 (2) Å
*c* = 12.5241 (3) Å
α = 88.386 (1)°
β = 70.743 (1)°
γ = 81.234 (2)°
*V* = 1260.48 (5) Å^3^
*Z* = 2
Mo *K*α radiation
μ = 0.32 mm^−1^
*T* = 100 (2) K
0.06 × 0.04 × 0.02 mm

#### Data collection

Bruker SMART APEX diffractometer
Absorption correction: multi-scan (*SADABS*; Sheldrick, 1996 ▶) *T*_min_ = 0.981, *T*_max_ = 0.994
12201 measured reflections
5762 independent reflections
4104 reflections with *I* > 2σ(*I*)
*R*_int_ = 0.032

#### Refinement

*R*[*F* ^2^ > 2σ(*F* ^2^)] = 0.048
*wR*(*F* ^2^) = 0.126
*S* = 1.03
5762 reflections
356 parameters
5 restraints
H atoms treated by a mixture of independent and constrained refinement
Δρ_max_ = 0.72 e Å^−3^
Δρ_min_ = −0.46 e Å^−3^

### 

Data collection: *APEX2* (Bruker, 2007 ▶); cell refinement: *SAINT* (Bruker, 2007 ▶); data reduction: *SAINT*; program(s) used to solve structure: *SHELXS97* (Sheldrick, 2008 ▶); program(s) used to refine structure: *SHELXL97* (Sheldrick, 2008 ▶); molecular graphics: *X-SEED* (Barbour, 2001 ▶); software used to prepare material for publication: *publCIF* (Westrip, 2009 ▶).

## Supplementary Material

Crystal structure: contains datablocks global, I. DOI: 10.1107/S1600536809002943/bt2856sup1.cif

Structure factors: contains datablocks I. DOI: 10.1107/S1600536809002943/bt2856Isup2.hkl

Additional supplementary materials:  crystallographic information; 3D view; checkCIF report

## Figures and Tables

**Table 1:** Hydrogen-bond geometry (Å, °)

*D*—H⋯*A*	*D*—H	H⋯*A*	*D*⋯*A*	*D*—H⋯*A*
O2—H21⋯N2	0.85 (1)	1.68 (2)	2.493 (2)	159 (4)
N1—H11⋯O1	0.89 (1)	1.73 (2)	2.520 (2)	147 (3)
N4—H41⋯O1	0.88 (1)	1.88 (1)	2.742 (2)	168 (2)
N4—H42⋯O3	0.89 (1)	1.89 (1)	2.769 (3)	169 (3)
N4—H43⋯O4^i^	0.89 (1)	1.92 (2)	2.755 (3)	154 (3)

Symmetry code: (i) 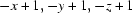.

**Fig. 1.**
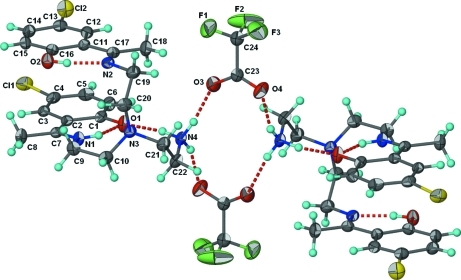
Anisotropic displacement ellipsoid plot (Barbour, 2001) of the title compound at the 70% probability level. H atoms are drawn as spheres of arbitrary radius.

## supplementary crystallographic information

### Experimental

Tris(2-aminoethyl)amine (1.46 g m 10 mmol) was condensed with 5-chloro-2-hydroxyacetophenone (5.12 g, 30 mol) in refluxing ethanol (100 ml) to yield the unsolvated Schiff base. The compound (0.64 g, 1 mmol) and trifluoroacetic acid (0.11 g, 1 mmol) were dissolved in a small volume of ethanol. Crystals of the salt separated after several days.

### Refinement

Carbon-bound H atoms were placed in calculated positions (C—H = 0.93–0.99 Å) and were included in the refinement in the riding model approximation, with *U*(H) set to 1.2 to 1.5*U*(C). The methyl H atoms were rotated to fit the electron density.

The iminium/ammonium and hydroxy H atoms were located in a difference Fourier map, and were refined with distance restraints [N—H = 0.88 (1) and O—H = 0.84 (1) Å; their isotropic displacement parameters were freely refined.

### Crystal data

**Table:**

C_22_H_29_Cl_2_N_4_O_2_^+^·C_2_F_3_O_2_^−^	*Z* = 2
*M**_r_* = 565.41	*F*(000) = 588
Triclinic, *P*1	*D*_x_ = 1.490 Mg m^−^^3^
Hall symbol: -P 1	Mo *K*α radiation, λ = 0.71073 Å
*a* = 10.1041 (2) Å	Cell parameters from 2402 reflections
*b* = 10.6788 (2) Å	θ = 2.2–27.8°
*c* = 12.5241 (3) Å	µ = 0.32 mm^−^^1^
α = 88.386 (1)°	*T* = 100 K
β = 70.743 (1)°	Prism, yellow
γ = 81.234 (2)°	0.06 × 0.04 × 0.02 mm
*V* = 1260.48 (5) Å^3^	

### Data collection

**Table:**

Bruker SMART APEX diffractometer	5762 independent reflections
Radiation source: fine-focus sealed tube	4104 reflections with *I* > 2σ(*I*)
graphite	*R*_int_ = 0.032
ω scans	θ_max_ = 27.5°, θ_min_ = 1.7°
Absorption correction: multi-scan (*SADABS*; Sheldrick, 1996)	*h* = −13→13
*T*_min_ = 0.981, *T*_max_ = 0.994	*k* = −13→13
12201 measured reflections	*l* = −16→16

### Refinement

**Table:**

Refinement on *F*^2^	Primary atom site location: structure-invariant direct methods
Least-squares matrix: full	Secondary atom site location: difference Fourier map
*R*[*F*^2^ > 2σ(*F*^2^)] = 0.048	Hydrogen site location: inferred from neighbouring sites
*wR*(*F*^2^) = 0.126	H atoms treated by a mixture of independent and constrained refinement
*S* = 1.02	*w* = 1/[σ^2^(*F*_o_^2^) + (0.0533*P*)^2^ + 0.6261*P*] where *P* = (*F*_o_^2^ + 2*F*_c_^2^)/3
5762 reflections	(Δ/σ)_max_ = 0.001
356 parameters	Δρ_max_ = 0.72 e Å^−^^3^
5 restraints	Δρ_min_ = −0.46 e Å^−^^3^

### Fractional atomic coordinates and isotropic or equivalent isotropic displacement parameters (Å^2^)

**Table:**

	*x*	*y*	*z*	*U*_iso_*/*U*_eq_	
Cl1	0.10326 (7)	0.47757 (6)	1.36535 (5)	0.03022 (16)	
Cl2	0.46582 (7)	0.29988 (6)	1.26264 (5)	0.03337 (17)	
O1	0.24624 (17)	0.42151 (15)	0.87521 (13)	0.0261 (4)	
O2	0.22931 (18)	−0.00604 (16)	1.01009 (14)	0.0258 (4)	
H21	0.278 (4)	0.005 (4)	0.9419 (14)	0.092 (14)*	
O3	0.6309 (2)	0.30567 (19)	0.55587 (16)	0.0445 (5)	
O4	0.7436 (2)	0.30693 (16)	0.36975 (14)	0.0349 (4)	
N1	0.0653 (2)	0.27355 (18)	0.90939 (15)	0.0203 (4)	
H11	0.135 (2)	0.316 (2)	0.871 (2)	0.042 (8)*	
N2	0.39560 (19)	0.06404 (17)	0.83106 (15)	0.0214 (4)	
N3	0.23242 (19)	0.19477 (17)	0.68450 (15)	0.0207 (4)	
N4	0.3612 (2)	0.4419 (2)	0.64538 (17)	0.0242 (4)	
H41	0.332 (3)	0.425 (2)	0.7177 (10)	0.027 (7)*	
H42	0.4511 (14)	0.408 (2)	0.613 (2)	0.044 (8)*	
H43	0.353 (3)	0.5260 (10)	0.641 (2)	0.038 (8)*	
C1	0.2076 (2)	0.4375 (2)	0.98379 (18)	0.0200 (5)	
C2	0.0963 (2)	0.37800 (19)	1.06063 (18)	0.0177 (4)	
C3	0.0633 (2)	0.3952 (2)	1.17817 (18)	0.0193 (5)	
H3	−0.0113	0.3569	1.2290	0.023*	
C4	0.1376 (2)	0.4664 (2)	1.21953 (18)	0.0216 (5)	
C5	0.2434 (2)	0.5277 (2)	1.1471 (2)	0.0247 (5)	
H5	0.2925	0.5787	1.1773	0.030*	
C6	0.2769 (2)	0.5144 (2)	1.0326 (2)	0.0246 (5)	
H6	0.3485	0.5578	0.9842	0.030*	
C7	0.0240 (2)	0.2942 (2)	1.01801 (18)	0.0179 (4)	
C8	−0.0912 (2)	0.2298 (2)	1.09553 (19)	0.0250 (5)	
H8A	−0.1262	0.1773	1.0509	0.037*	
H8B	−0.1692	0.2938	1.1394	0.037*	
H8C	−0.0539	0.1763	1.1471	0.037*	
C9	0.0156 (2)	0.1860 (2)	0.84896 (19)	0.0227 (5)	
H9A	−0.0893	0.2016	0.8730	0.027*	
H9B	0.0458	0.0976	0.8668	0.027*	
C10	0.0773 (2)	0.2056 (2)	0.72269 (19)	0.0231 (5)	
H10A	0.0499	0.1418	0.6814	0.028*	
H10B	0.0368	0.2906	0.7044	0.028*	
C11	0.3915 (2)	0.1380 (2)	1.00730 (18)	0.0193 (5)	
C12	0.4442 (2)	0.2098 (2)	1.07147 (19)	0.0214 (5)	
H12	0.5183	0.2569	1.0342	0.026*	
C13	0.3905 (2)	0.2132 (2)	1.18738 (19)	0.0222 (5)	
C14	0.2819 (2)	0.1461 (2)	1.24520 (19)	0.0237 (5)	
H14	0.2442	0.1503	1.3255	0.028*	
C15	0.2300 (2)	0.0732 (2)	1.18385 (19)	0.0224 (5)	
H15	0.1565	0.0261	1.2227	0.027*	
C16	0.2827 (2)	0.0670 (2)	1.06586 (19)	0.0208 (5)	
C17	0.4476 (2)	0.1367 (2)	0.88267 (19)	0.0214 (5)	
C18	0.5569 (3)	0.2198 (2)	0.8265 (2)	0.0273 (5)	
H18A	0.5891	0.2051	0.7444	0.041*	
H18B	0.6377	0.1995	0.8540	0.041*	
H18C	0.5152	0.3089	0.8443	0.041*	
C19	0.4354 (3)	0.0499 (2)	0.70858 (19)	0.0259 (5)	
H19A	0.4932	−0.0341	0.6833	0.031*	
H19B	0.4927	0.1159	0.6711	0.031*	
C20	0.3015 (2)	0.0623 (2)	0.6765 (2)	0.0247 (5)	
H20A	0.3258	0.0295	0.5981	0.030*	
H20B	0.2347	0.0104	0.7273	0.030*	
C21	0.2872 (3)	0.2563 (2)	0.57570 (19)	0.0266 (5)	
H21A	0.2368	0.2340	0.5249	0.032*	
H21B	0.3889	0.2223	0.5404	0.032*	
C22	0.2712 (3)	0.3986 (2)	0.5850 (2)	0.0274 (5)	
H22A	0.2968	0.4336	0.5081	0.033*	
H22B	0.1706	0.4325	0.6256	0.033*	
C23	0.7267 (2)	0.2710 (2)	0.4670 (2)	0.0245 (5)	
C24	0.8459 (3)	0.1700 (2)	0.4815 (2)	0.0281 (5)	
F1	0.79561 (19)	0.07794 (17)	0.54812 (16)	0.0644 (6)	
F2	0.9303 (2)	0.21998 (18)	0.5210 (2)	0.0813 (8)	
F3	0.92729 (18)	0.11073 (17)	0.38502 (15)	0.0550 (5)	

### Atomic displacement parameters (Å^2^)

**Table:**

	*U*^11^	*U*^22^	*U*^33^	*U*^12^	*U*^13^	*U*^23^
Cl1	0.0342 (3)	0.0384 (4)	0.0199 (3)	0.0023 (3)	−0.0144 (3)	−0.0062 (2)
Cl2	0.0386 (4)	0.0377 (4)	0.0288 (3)	−0.0110 (3)	−0.0150 (3)	−0.0050 (3)
O1	0.0300 (9)	0.0305 (9)	0.0170 (8)	−0.0130 (7)	−0.0031 (7)	0.0024 (7)
O2	0.0267 (9)	0.0292 (9)	0.0234 (9)	−0.0125 (7)	−0.0069 (8)	0.0015 (7)
O3	0.0322 (10)	0.0547 (13)	0.0297 (10)	0.0114 (9)	0.0037 (8)	0.0106 (9)
O4	0.0475 (11)	0.0331 (10)	0.0223 (9)	−0.0028 (8)	−0.0111 (8)	0.0062 (7)
N1	0.0228 (10)	0.0221 (10)	0.0169 (9)	−0.0087 (8)	−0.0054 (8)	−0.0002 (7)
N2	0.0216 (10)	0.0239 (10)	0.0186 (9)	−0.0002 (8)	−0.0078 (8)	0.0011 (8)
N3	0.0235 (10)	0.0230 (10)	0.0149 (9)	−0.0012 (8)	−0.0065 (8)	−0.0002 (7)
N4	0.0251 (11)	0.0292 (12)	0.0153 (10)	−0.0029 (9)	−0.0034 (9)	0.0054 (8)
C1	0.0233 (11)	0.0173 (11)	0.0197 (11)	−0.0029 (9)	−0.0076 (9)	0.0027 (8)
C2	0.0183 (11)	0.0156 (10)	0.0199 (11)	−0.0020 (8)	−0.0075 (9)	0.0006 (8)
C3	0.0202 (11)	0.0190 (11)	0.0165 (11)	0.0015 (9)	−0.0050 (9)	0.0007 (8)
C4	0.0269 (12)	0.0216 (11)	0.0174 (11)	0.0016 (9)	−0.0110 (10)	−0.0022 (9)
C5	0.0286 (12)	0.0202 (12)	0.0312 (13)	−0.0046 (9)	−0.0170 (11)	−0.0014 (9)
C6	0.0268 (12)	0.0207 (12)	0.0282 (13)	−0.0095 (9)	−0.0094 (10)	0.0035 (9)
C7	0.0191 (11)	0.0159 (10)	0.0186 (11)	−0.0005 (8)	−0.0071 (9)	0.0003 (8)
C8	0.0282 (12)	0.0281 (13)	0.0196 (11)	−0.0121 (10)	−0.0058 (10)	0.0021 (9)
C9	0.0248 (12)	0.0242 (12)	0.0215 (11)	−0.0074 (9)	−0.0088 (10)	−0.0031 (9)
C10	0.0271 (12)	0.0241 (12)	0.0203 (11)	−0.0028 (9)	−0.0110 (10)	−0.0021 (9)
C11	0.0205 (11)	0.0179 (11)	0.0191 (11)	−0.0013 (9)	−0.0067 (9)	0.0018 (8)
C12	0.0200 (11)	0.0190 (11)	0.0255 (12)	−0.0039 (9)	−0.0075 (10)	0.0032 (9)
C13	0.0236 (12)	0.0211 (12)	0.0237 (12)	−0.0011 (9)	−0.0108 (10)	−0.0017 (9)
C14	0.0245 (12)	0.0256 (12)	0.0179 (11)	0.0009 (9)	−0.0050 (9)	0.0011 (9)
C15	0.0200 (11)	0.0212 (11)	0.0231 (12)	−0.0034 (9)	−0.0033 (9)	0.0034 (9)
C16	0.0179 (11)	0.0207 (11)	0.0234 (12)	−0.0015 (9)	−0.0069 (9)	0.0012 (9)
C17	0.0192 (11)	0.0199 (11)	0.0251 (12)	−0.0013 (9)	−0.0083 (10)	0.0029 (9)
C18	0.0277 (13)	0.0311 (13)	0.0216 (12)	−0.0091 (10)	−0.0046 (10)	0.0049 (10)
C19	0.0283 (13)	0.0291 (13)	0.0182 (11)	0.0023 (10)	−0.0075 (10)	−0.0010 (9)
C20	0.0302 (13)	0.0238 (12)	0.0209 (12)	−0.0009 (10)	−0.0106 (10)	−0.0035 (9)
C21	0.0310 (13)	0.0331 (13)	0.0150 (11)	−0.0018 (10)	−0.0078 (10)	0.0008 (9)
C22	0.0313 (13)	0.0306 (13)	0.0209 (12)	−0.0048 (10)	−0.0097 (10)	0.0075 (10)
C23	0.0244 (12)	0.0243 (12)	0.0239 (12)	−0.0046 (9)	−0.0069 (10)	0.0038 (9)
C24	0.0271 (13)	0.0301 (13)	0.0257 (13)	−0.0042 (10)	−0.0072 (11)	0.0029 (10)
F1	0.0543 (11)	0.0495 (11)	0.0659 (13)	0.0096 (9)	0.0014 (10)	0.0328 (10)
F2	0.0898 (15)	0.0505 (12)	0.143 (2)	0.0103 (10)	−0.0980 (16)	−0.0211 (12)
F3	0.0445 (10)	0.0601 (12)	0.0440 (10)	0.0166 (8)	−0.0026 (8)	−0.0095 (8)

### Geometric parameters (Å, °)

**Table:**

Cl1—C4	1.747 (2)	C9—C10	1.518 (3)
Cl2—C13	1.749 (2)	C9—H9A	0.9900
O1—C1	1.294 (3)	C9—H9B	0.9900
O2—C16	1.339 (3)	C10—H10A	0.9900
O2—H21	0.847 (10)	C10—H10B	0.9900
O3—C23	1.231 (3)	C11—C12	1.400 (3)
O4—C23	1.231 (3)	C11—C16	1.419 (3)
N1—C7	1.299 (3)	C11—C17	1.474 (3)
N1—C9	1.460 (3)	C12—C13	1.371 (3)
N1—H11	0.889 (10)	C12—H12	0.9500
N2—C17	1.293 (3)	C13—C14	1.389 (3)
N2—C19	1.458 (3)	C14—C15	1.377 (3)
N3—C10	1.467 (3)	C14—H14	0.9500
N3—C20	1.469 (3)	C15—C16	1.396 (3)
N3—C21	1.469 (3)	C15—H15	0.9500
N4—C22	1.487 (3)	C17—C18	1.501 (3)
N4—H41	0.878 (10)	C18—H18A	0.9800
N4—H42	0.886 (10)	C18—H18B	0.9800
N4—H43	0.892 (10)	C18—H18C	0.9800
C1—C6	1.423 (3)	C19—C20	1.520 (3)
C1—C2	1.436 (3)	C19—H19A	0.9900
C2—C3	1.409 (3)	C19—H19B	0.9900
C2—C7	1.450 (3)	C20—H20A	0.9900
C3—C4	1.365 (3)	C20—H20B	0.9900
C3—H3	0.9500	C21—C22	1.507 (3)
C4—C5	1.391 (3)	C21—H21A	0.9900
C5—C6	1.367 (3)	C21—H21B	0.9900
C5—H5	0.9500	C22—H22A	0.9900
C6—H6	0.9500	C22—H22B	0.9900
C7—C8	1.494 (3)	C23—C24	1.545 (3)
C8—H8A	0.9800	C24—F2	1.299 (3)
C8—H8B	0.9800	C24—F1	1.322 (3)
C8—H8C	0.9800	C24—F3	1.329 (3)
			
C16—O2—H21	102 (3)	C13—C12—H12	119.5
C7—N1—C9	127.14 (19)	C11—C12—H12	119.5
C7—N1—H11	113.5 (19)	C12—C13—C14	121.3 (2)
C9—N1—H11	119.3 (19)	C12—C13—Cl2	118.69 (18)
C17—N2—C19	124.9 (2)	C14—C13—Cl2	119.96 (18)
C10—N3—C20	112.17 (18)	C15—C14—C13	118.7 (2)
C10—N3—C21	111.60 (18)	C15—C14—H14	120.7
C20—N3—C21	109.93 (18)	C13—C14—H14	120.7
C22—N4—H41	112.4 (17)	C14—C15—C16	121.5 (2)
C22—N4—H42	110.4 (19)	C14—C15—H15	119.2
H41—N4—H42	110 (3)	C16—C15—H15	119.2
C22—N4—H43	107.7 (18)	O2—C16—C15	119.2 (2)
H41—N4—H43	106 (2)	O2—C16—C11	121.3 (2)
H42—N4—H43	109 (3)	C15—C16—C11	119.5 (2)
O1—C1—C6	120.6 (2)	N2—C17—C11	116.1 (2)
O1—C1—C2	122.6 (2)	N2—C17—C18	125.6 (2)
C6—C1—C2	116.8 (2)	C11—C17—C18	118.3 (2)
C3—C2—C1	119.7 (2)	C17—C18—H18A	109.5
C3—C2—C7	119.8 (2)	C17—C18—H18B	109.5
C1—C2—C7	120.32 (19)	H18A—C18—H18B	109.5
C4—C3—C2	120.5 (2)	C17—C18—H18C	109.5
C4—C3—H3	119.8	H18A—C18—H18C	109.5
C2—C3—H3	119.8	H18B—C18—H18C	109.5
C3—C4—C5	121.0 (2)	N2—C19—C20	108.94 (19)
C3—C4—Cl1	119.76 (18)	N2—C19—H19A	109.9
C5—C4—Cl1	119.19 (17)	C20—C19—H19A	109.9
C6—C5—C4	120.0 (2)	N2—C19—H19B	109.9
C6—C5—H5	120.0	C20—C19—H19B	109.9
C4—C5—H5	120.0	H19A—C19—H19B	108.3
C5—C6—C1	121.9 (2)	N3—C20—C19	111.49 (19)
C5—C6—H6	119.0	N3—C20—H20A	109.3
C1—C6—H6	119.0	C19—C20—H20A	109.3
N1—C7—C2	117.78 (19)	N3—C20—H20B	109.3
N1—C7—C8	120.4 (2)	C19—C20—H20B	109.3
C2—C7—C8	121.75 (19)	H20A—C20—H20B	108.0
C7—C8—H8A	109.5	N3—C21—C22	114.00 (19)
C7—C8—H8B	109.5	N3—C21—H21A	108.8
H8A—C8—H8B	109.5	C22—C21—H21A	108.8
C7—C8—H8C	109.5	N3—C21—H21B	108.8
H8A—C8—H8C	109.5	C22—C21—H21B	108.8
H8B—C8—H8C	109.5	H21A—C21—H21B	107.6
N1—C9—C10	109.14 (18)	N4—C22—C21	112.88 (19)
N1—C9—H9A	109.9	N4—C22—H22A	109.0
C10—C9—H9A	109.9	C21—C22—H22A	109.0
N1—C9—H9B	109.9	N4—C22—H22B	109.0
C10—C9—H9B	109.9	C21—C22—H22B	109.0
H9A—C9—H9B	108.3	H22A—C22—H22B	107.8
N3—C10—C9	112.01 (18)	O3—C23—O4	130.0 (2)
N3—C10—H10A	109.2	O3—C23—C24	114.3 (2)
C9—C10—H10A	109.2	O4—C23—C24	115.6 (2)
N3—C10—H10B	109.2	F2—C24—F1	109.2 (2)
C9—C10—H10B	109.2	F2—C24—F3	106.2 (2)
H10A—C10—H10B	107.9	F1—C24—F3	104.2 (2)
C12—C11—C16	118.0 (2)	F2—C24—C23	111.5 (2)
C12—C11—C17	120.8 (2)	F1—C24—C23	112.3 (2)
C16—C11—C17	121.2 (2)	F3—C24—C23	113.1 (2)
C13—C12—C11	121.0 (2)		
			
O1—C1—C2—C3	177.41 (19)	Cl2—C13—C14—C15	−176.52 (17)
C6—C1—C2—C3	−1.5 (3)	C13—C14—C15—C16	−0.8 (3)
O1—C1—C2—C7	1.2 (3)	C14—C15—C16—O2	179.5 (2)
C6—C1—C2—C7	−177.62 (19)	C14—C15—C16—C11	−0.6 (3)
C1—C2—C3—C4	−1.0 (3)	C12—C11—C16—O2	−178.6 (2)
C7—C2—C3—C4	175.23 (19)	C17—C11—C16—O2	1.6 (3)
C2—C3—C4—C5	2.6 (3)	C12—C11—C16—C15	1.5 (3)
C2—C3—C4—Cl1	−175.57 (16)	C17—C11—C16—C15	−178.3 (2)
C3—C4—C5—C6	−1.7 (3)	C19—N2—C17—C11	179.43 (19)
Cl1—C4—C5—C6	176.49 (18)	C19—N2—C17—C18	0.4 (4)
C4—C5—C6—C1	−0.9 (4)	C12—C11—C17—N2	177.7 (2)
O1—C1—C6—C5	−176.5 (2)	C16—C11—C17—N2	−2.5 (3)
C2—C1—C6—C5	2.4 (3)	C12—C11—C17—C18	−3.2 (3)
C9—N1—C7—C2	175.0 (2)	C16—C11—C17—C18	176.6 (2)
C9—N1—C7—C8	−3.1 (3)	C17—N2—C19—C20	−131.8 (2)
C3—C2—C7—N1	−174.86 (19)	C10—N3—C20—C19	−145.03 (19)
C1—C2—C7—N1	1.3 (3)	C21—N3—C20—C19	90.2 (2)
C3—C2—C7—C8	3.3 (3)	N2—C19—C20—N3	74.7 (2)
C1—C2—C7—C8	179.4 (2)	C10—N3—C21—C22	78.2 (2)
C7—N1—C9—C10	172.3 (2)	C20—N3—C21—C22	−156.73 (19)
C20—N3—C10—C9	76.3 (2)	N3—C21—C22—N4	67.2 (3)
C21—N3—C10—C9	−159.79 (18)	O3—C23—C24—F2	−76.8 (3)
N1—C9—C10—N3	54.4 (2)	O4—C23—C24—F2	101.3 (3)
C16—C11—C12—C13	−1.1 (3)	O3—C23—C24—F1	46.1 (3)
C17—C11—C12—C13	178.72 (19)	O4—C23—C24—F1	−135.8 (2)
C11—C12—C13—C14	−0.2 (3)	O3—C23—C24—F3	163.6 (2)
C11—C12—C13—Cl2	177.53 (17)	O4—C23—C24—F3	−18.3 (3)
C12—C13—C14—C15	1.2 (3)		

### Hydrogen-bond geometry (Å, °)

**Table:**

*D*—H···*A*	*D*—H	H···*A*	*D*···*A*	*D*—H···*A*
O2—H21···N2	0.85 (1)	1.68 (2)	2.493 (2)	159 (4)
N1—H11···O1	0.89 (1)	1.73 (2)	2.520 (2)	147 (3)
N4—H41···O1	0.88 (1)	1.88 (1)	2.742 (2)	168 (2)
N4—H42···O3	0.89 (1)	1.89 (1)	2.769 (3)	169 (3)
N4—H43···O4^i^	0.89 (1)	1.92 (2)	2.755 (3)	154 (3)

Symmetry codes: (i) −*x*+1, −*y*+1, −*z*+1.

## References

[bb1] Barbour, L. J. (2001). *J. Supramol. Chem.* **1**, 189–191.

[bb2] Bruker (2007). *APEX2* and *SAINT* Bruker AXS Inc., Madison, Wisconsin, USA.

[bb3] Lee, S. M., Ali, H. M., Lo, K. M. & Ng, S. W. (2009). *Acta Cryst.* E65, o409. 10.1107/S1600536809002906 PMC2968323 21582001

[bb4] Sheldrick, G. M. (1996). *SADABS* University of Göttingen, Germany.

[bb5] Sheldrick, G. M. (2008). *Acta Cryst.* A**64**, 112–122. 10.1107/S0108767307043930 18156677

[bb6] Westrip, S. P. (2009). *publCIF* In preparation.

